# Topologies of power in China’s grid-style social management during the COVID-19 pandemic

**DOI:** 10.1177/09670106221134968

**Published:** 2022-12-06

**Authors:** Sabrina Habich-Sobiegalla, Franziska Plümmer

**Affiliations:** Freie Universität Berlin, Germany; University of Amsterdam, Netherlands

**Keywords:** China, COVID-19, Foucault, grid-style social management, power, security apparatus

## Abstract

This article analyses the organization of Chinese grassroots social management during the COVID-19 pandemic. Drawing on a range of local cases researched through policy documents, media coverage and interviews, we scrutinize the appropriation of emergency measures and the utilization of grid-style social management since the outbreak of COVID-19. Grid-style social management – a new grassroots administrative division aiming to mobilize neighbourhood control and services – is a core element in China’s pursuit of economic growth without sacrificing political stability. Conceptualizing grids as confined spaces of power, we show how the Chinese party-state is able to flexibly redeploy diverse forms of power depending on the particular purpose of social management. During non-crisis times, grid-style social management primarily uses security power, casting a net over the population that remains open for population elements to contribute their share to the national economy. Once a crisis has been called, sovereign power swiftly closes the net to prevent further circulation while disciplinary power works towards a speedy return to a pre-crisis routine.

## Introduction

Staying still and starting to move!

Pushing the pause button and pushing the start button!

Falling data and a stable economy!

We demand strict obedience. Under the same baton, there is a kind of mutual guard called ‘fighting the epidemic side by side’: a million people of Zhuji walk together!

Both hands have to be firm; both wars have to be won!

Fighting to get back the time wasted by the epidemic!

Taking back the profit lost through the epidemic!

*Zhejiang News*, 3 March 2020

The issue of mobility control and territorial governmentality is now a cornerstone of critical security studies. Probing the myriad intersections between mobility, security practices and population management, previous research has scrutinized spatial control of individual mobility in refugee camps (Bulley, 2014), mobility management of ‘dangerous populations’ (Berda, 2020), international mobility control with digital means (Glouftsios, 2021; Pallister-Wilkins, 2016) and concepts of mobility and circulation in regional spatial planning (Moisio and Luukkonen, 2015). With the global proliferation of previously ‘unthinkable’ spatial tools such as (border) lockdowns, contact tracing and social distancing rules, the recent COVID-19 pandemic provides the opportunity to expand this literature (Boin et al, 2021).

In many democratic societies, the appropriateness and legitimacy of state intervention during the pandemic have been under constant negotiation (Schmidt, 2021). In an authoritarian system such as China’s that builds on a history of socialist planning, pandemic measures met a more expansive set of spatial tools than previously assumed. To better understand the rationalities behind these tools and their effect on mobility in China during the COVID-19 pandemic, this article scrutinizes a spatialized security system – grid-style social management (GSM, *wanggeshehui guanli*) – at the heart of managing mobility during the COVID-19 outbreak in China. In detail, we ask how the Chinese party-state used GSM to contain the spread of COVID-19 during the first half of 2020. We show that the party-state tightened the already existing spatialized security system of GSM to manage states of gridlock and mobility among different communities. This allowed the Chinese government to move quickly from lockdowns as emergency measures to the mobilization of society for the sake of the economy. It also allowed the party-state to desecuritize the pandemic soon after the outbreak was first acknowledged and to use the crisis ‘as opportunit[y] to be seized to transform various aspects of social reality’ (Prozorov, 2021: 436).

First introduced in Beijing’s Dongcheng district in 2004, grids were established by local governments across China to improve street-level policing and service provision in their jurisdictions (Wu, 2014). A grid constitutes an administrative unit that is built upon previously existing resident groups (*jumin xiaozu* in urban China) or villager groups (*cunmin xiaozu* in rural China) one level below the community (urban) or village (rural) level (Xu and He, 2022).^1^ While the size and organization of each grid differs from place to place, Tang (2020) reports that in urban middle-class communities, grids cover around 600 households. In rural areas, this number may be substantially lower (Interview 5). On the surface, GSM introduces what the party-state refers to as co-governance (*gongzhi*), where various actors (i.e. government officials, volunteers, representatives of social organizations – so-called grid managers) cooperate in implementing government initiatives (Tang, 2020). GSM thereby decentralizes social management by giving grassroots institutions the responsibility of self-regulation. In essence, however, this self-regulation takes place under the guidance of local governments, who, as the superiors of grid managers, can intervene as they see fit. At the same time, it introduces new registers in the form of local digital platforms that store residents’ information living in each grid (Hu, 2013).

Although GSM existed prior to the COVID-19 pandemic, we regard grids as spatial articulations of the Chinese security apparatus that, during the pandemic, allowed local governments to relaunch their economies without risking extensive rises in infections. This spatial organization represents a core element in China’s concurrent pursuit of economic growth and social stability, reflecting a continuous desire to secure the circulation of different population elements. As mentioned in the poem cited above from Zhuji city (Zhejiang province), the Chinese *fight against the epidemic* builds on a collective effort implemented through GSM. This effort was founded upon the creation of sociospatial boundaries to prevent the further circulation of the virus (*People’s Daily*, 2020).

We follow Foucault (2009) and Collier (2009) in undertaking a topological analysis of the grid. Foucault distinguishes between disciplinary spaces and spaces of security (also referred to as milieux). While the former is ‘artificial and constraining [. . .] work[ing] in a sphere complimentary to reality’ (such as schools or prisons), spaces of security ‘work within reality’ by utilizing natural and artificial givens to ensure orderly and secure circulations of all population elements (Foucault, 2009: 47). We regard the grid as both a disciplinary space and a milieu, depending on the primary purpose of governing at a particular time. For example, in non-crisis times, grid borders are permeable, allowing mobility of grid residents while ensuring that mobility does not engender instability. The primary function thus builds on security technologies that plan and anticipate, avoiding crisis while also deploying disciplinary technologies within grid borders to educate and ‘stabilize’ grid residents. In times of crisis, grid borders become almost impermeable. As sovereign and disciplinary power come to the fore to enforce lockdowns and monitor individual behaviour (and, in this case, personal health), planning and anticipation (i.e. security technologies) move to the background.

By utilizing a spatial reading of power to scrutinize the intersection of social management and crisis politics in China, this article makes the following theoretical and empirical contributions: first, by detailing the security technologies present in authoritarian China’s pandemic management, our analysis contributes to the critical security studies literature that discusses ‘critical mobilities’ (Cresswell, 2014; Söderström et al, 2013) and the role of surveillance technologies in governing mobility and related infrastructures (Bell, 2016; Glouftsios, 2021) which has, thus far, almost exclusively focused on liberal regimes. Secondly, by discussing how the Chinese government utilizes its security practices in times of crisis, this article also contributes theoretically to the literature on authoritarian governmentality (Jeffreys and Sigley, 2009; Palmer and Winiger, 2019) and its explanation of state-society relations (Habich-Sobiegalla and Plümmer, 2021; Habich-Sobiegalla and Rousseau, 2020). The article further speaks to the governance debate on China’s economic and political transition, in which scholars such as Heilmann (2017) argue that the party-state switches between a transition mode (characterized by relatively liberal politics) and a crisis mode (in which the party-state recentralizes authority, reemphasizes ideology and rigidly enforces measures). We show that these modes blend, meaning that crisis anticipation and management are part of overall social management. Finally, we provide an in-depth analysis of China’s social management during the COVID-19 pandemic contributing to a seminal debate on China’s pandemic management (Ahmad, 2022; Li et al, 2021; Xu and He, 2022; Yao et al, 2020).

Methodologically, this article builds on a convenience sample of local cases that illustrate the implementation of anti-epidemic measures through GSM. Being scholars based outside of China, we have not been able to resume fieldwork since the outbreak of COVID-19. However, we continue our investigation from a distance by drawing data from policy documents, media coverage of anti-epidemic measures, discussions with municipal officials responsible for grassroots governance and online interviews with grid managers, community volunteers and academics. The interviews were held via WeChat, some by us and some by a Chinese research assistant. Given the increased personal risk of journalists and informants cooperating with foreign scholars on ‘sensitive’ issues, we discussed possible repercussions and ‘red lines’ with the interviewees (Glasius et al, 2018), accepted refusals and anonymized their contributions. The article should therefore not be regarded as definitive but rather as a first heuristic probe into the rationalities and potential consequences of China’s GSM during the COVID-19 pandemic of 2020 and the evolution of the Chinese security apparatus.

The remainder of this article is organized as follows: the subsequent section introduces Foucault’s spatial topology of power and its application to GSM as a mechanism to carefully regulate the circulation of mobility within the Chinese security system. We discuss how governing in China functions through a lens of (neosocialist) governmentality (see Palmer and Winiger, 2019), which we argue builds on individual and collective strategies of control operationalized through GSM and a specific combination of sovereign, disciplinary and security power. In the following two sections, we investigate how during the first outbreak of COVID-19 in 2020, the government used GSM to deploy different types of power depending on the particular purpose of social management. While during the first phase of general lockdowns, sovereignty and discipline dominated China’s neosocialist governmentality, the second phase of reopening and targeted lockdowns was driven by security and discipline. In the conclusion, we discuss how handling this health crisis reveals the continuous efforts by China’s security apparatus to govern through spatial technologies of social management.

## Topologies of power in China

Michel Foucault’s concept of power explores how the government operates through the individual subject by simultaneously employing three different forms of power. First, sovereign power is the power a government exercises over its territory and subjects through laws that define the citizen and differentiate between different groups of citizens. Second, disciplinary power aims to control the individual through institutions such as education or incarceration. And third, security – also referred to as regulatory power – undertakes ‘modulated interventions into the field of autonomous and mutually corrective decisions’ to affect a population (Collier, 2009: 87). We thus follow what Collier (2009) has termed a topological analysis of power prevalent in Foucault’s later lectures on *Territory, Security, Population*. Rather than assuming a single logic of power in a given period, such an analysis reveals how distinct forms of power recombine in a ‘topological space’ – ‘in different sectors, at a given moment, in a given society, in a given country’ (Collier, 2009: 90; Foucault, 2009: 8).

In line with the different technologies used, these three forms of power also reveal distinct treatments of space: sovereignty is exercised over territory with a spatial layout that allows for adequate policing to protect the territory and guarantee political circulations. Discipline works through institutions (or disciplinary spaces) artificially created to target the individual (Foucault, 2009: 15–16). Security affects the population through milieux (or spaces of security) that are confined by both material givens (such as rivers, hills and islands) and artificial givens (such as houses or agglomerations of individuals). Considering these artificial and material givens, security attempts to plan a milieu by anticipating how future events might unfold between ‘individuals, populations, and groups, and quasi-natural events which occur around them’ (2009: 21). To ensure a productive and orderly population, the freedom of the population and the circulation of objects is governed through technologies of security that structure and construct space in line with the ‘functional effects specific to this distribution, for example, ensuring trade, housing’ and hygiene (2009: 17).

Freedom of circulation and control through structure exist simultaneously and must constantly be negotiated. While being ‘an architect of the disciplined space’, the territorial sovereign is also ‘the regulator’ of milieux. In contrast to discipline, security is enacted not by ‘establishing limits and frontiers or fixing locations’ but by ensuring circulation and predicting possible events and adaptations of circulations within the given territory (Foucault, 2009: 51). When town planners create spaces such as cities, they must anticipate the kind of natural, artificial elements that will inhabit these spaces and what a desirable circulation among these elements should look like. Once a space is designed, the boundaries of milieux function as artificial barriers of circulation that can be opened and closed according to government rationale. Foucault (2009) uses the example of food scarcity to show how a specific event can quickly affect a specific milieu (in this case, the urban milieu), making it necessary for the sovereign to predict such events to avoid crises.

We argue that GSM represents a topology of all three forms of power that, depending on the primary purpose of governing (i.e. maintaining political stability or promoting economic growth), is shaped differently. First, sovereign power creates grids and borders to establish a spatial distribution in favour of political effectiveness, allowing the party-state an extended reach into local communities. In pre-crisis times, when goods and people have to circulate to achieve economic growth, grid borders are permeable and do not intervene in people’s daily lives. During the pandemic, sovereign power was most widely applied during the early stages after the official acknowledgement of the virus in China. This was when lockdowns were enforced all over the country, prohibiting people from leaving their residential compounds (or grids) and punishing transgressions of that rule. Second, grids constitute disciplinary spaces that, based on sovereign rule, supervise and control individuals even before a transgression has occurred (e.g. through written manuals and other re-education measures). In non-crisis times, the police aims to punish transgressive behaviour while grid managers are concerned with community-building (e.g. educating residents about socially acceptable behaviour as sanctioned by the party-state). During a crisis, grid managers increasingly permeate the public and private lives of their residents to ensure rule abidance. Finally, security tries to ensure mobility within and among grids. It is the dominant form of power in non-crisis times when it calculates and predicts the rate of offences in a population. It also takes centre stage when the emergency phase ends, and circulation of goods and people is to resume. Security power then calculates the overall cost for society of somebody leaving their compound during the pandemic and potentially spreading the virus and the threshold beyond which the cost of preventing the spread of the virus surpasses the cost of potential economic decline. Based on such calculations, necessary and appropriate crisis measures, such as when to deploy a lockdown and reopen, are determined. In this way, grids function as sociospatial interventions that balance mobility across time.

A topological analysis of power reveals the specific configurations of how population, power and territory interplay, allowing us to account for the spatial aspects of China’s grassroots social management and to identify the forms of power deployed through this spatial regime (see Table I). We can also distinguish between different periods in China’s crisis and population management as perceived by the political leadership. In detail, we identify a first phase of emergency measures that prevented all mobilities and a second phase that focused on resuming economic mobility and managing what Chinese state media have frequently referred to as the ‘twin wars’ (i.e. the parallel fights against the virus and economic decline) by selectively opening and closing grid borders. In the case of Zhejiang, for example, the local lockdown as part of the emergency measures was implemented on 2 February 2020, with relaxation of the measures beginning only six days later on 8 February (*CZTV*, 2020). While there are local differences regarding the exact dates that emergency measures and subsequent resumptions of work began, the techniques used were similar across China, no matter when precisely each phase began and how long it lasted (Yao et al, 2020). We argue that the power mix deployed through the grids in each phase (i.e. the emergency and ‘twin war’ phases) was also similar across regions in China, with some localities having undergone repetitive rounds of lockdowns and reopening.^2^ Considering the ongoing zero-Covid strategy, it is unknown if or when there ever will be a post-crisis mode.

**Table I.** Functioning of grids in crisis and non-crisis times.

Temporal regime	Sovereignty	Discipline	Security
Pre-crisis times	Territory: Delineate boundaries between grids	Policing of unwanted behaviour through patrols; community-building; community mobilization	Anticipate crisis, ensure circulation among grids to strengthen economic performance
Emergency phase	Mobility control: enforce lockdowns through border closures, and social distancing	Intense policing of unwanted behaviour through house visits and quarantine enforcement; educating the community through written manuals that assert logic of appropriateness, punishment and re-education	Work towards reopening through measures that ensure ‘secure’ circulation
Twin wars	Selectively manage grid boundaries	Less policing of unwanted behaviour through patrols; community-building; community mobilization	Heightened control through digital means, aims to anticipate new emergencies

Source: Authors’ own compilation based on Foucault (2009).

The literature consulting Foucault for studying authoritarian systems – especially China – has proliferated in recent years. Studies include critical analyses of the *hukou* (Chinese household registration) system (Wang and Liu, 2016; Zhang, 2018), birth planning (Greenhalgh and Winckler, 2005) and spatial planning and urban architecture (Dutton, 1992; Yang, 2011). Spatial articulations such as the work unit (*danwei*)^3^ (Bray, 2008) and the community (*shequ*) – an attempt to revitalize community engagement (Heberer and Göbel, 2011) – have also received academic interest. These studies show how socialist and imperial institutions of the pre-reform era continue to influence social management practices in contemporary China. For example, by differentiating citizens according to their place of birth and work through the *hukou* system, the government can link the mobilization of markets and migration across its territory (Ong, 2006). At the same time, collecting citizens’ personal information allows them to establish national statistics on population growth and other social indicators (Wang and Liu, 2016: 154), equipping the party-state with the ability to predict and influence circulation through administrative barriers. The Party and its members have been playing a vital role within this security apparatus, extending the governmental reach to the grassroots level and blending public and private spheres (Bray, 2008: 399).

Despite economic reforms and a growing – albeit limited – autonomy of subjects, the Chinese Communist Party (CCP) has been guiding individuals towards realizing collective goals and continues to govern through state intervention and social engineering (Jeffreys and Sigley, 2009: 7). While Jeffreys and Sigley (2009) see similarities in the technologies that liberal and illiberal regimes use to guide conduct, Palmer and Winiger (2019) argue for a distinct form of neosocialist governmentality which is characterized by local practices that build on a

vertically organized apparatus equipped with increasingly sophisticated instruments of social engineering and for shaping peoples’ subjectivities and guiding their conduct from a distance. [. . .] Indeed, this governmentality is *neo*-socialist in that its explicit aim is to revive socialism and open a new phase in socialist construction after the exhaustion of both the classical socialist command economy and the Maoist revolutionary mobilization. (Palmer and Winiger, 2019: 560)

At the same time, neosocialism conflates a market economy with the political goals of the CCP, in support of which it appropriates both illiberal and neoliberal techniques (Habich-Sobiegalla and Rousseau, 2020).

We provide further nuance to such conceptualizations of Chinese governmentality by looking at China’s grassroots health crisis management through a spatial–temporal lens. First, we identify different periods of health crisis management characterized by differences in the forms and combinations of power deployed. In so doing, we can show how, depending on the dominant political purpose of the time, different aspects of China’s governmentality manifest over time. While the first phase of emergency measures was dominated by socialist community mobilization techniques and authoritarian lockdowns, the second phase of the ‘twin wars’ saw the return of (neo)liberal forms of government. Second, we scrutinize a spatial aspect of China’s governmentality by showing how the spatial regime of GSM has allowed for the flexible adaptation of power assemblies. Hence, in contrast to previous studies which have highlighted different types of power and their consequences for subject formation in a given policy field such as health or migration, we lay bare how, over a short period, the topology of power within a single policy field may change with the help of (spatial) instruments of power (here: grids). Against this background, we argue that China’s governmentality aims to manage crises by enacting states of emergency and the long-term construction of mechanisms (such as GSM) that allow it to flexibly shut down and, in turn, reboot circulation.

## Spaces of discipline and security during COVID-19 grassroots epidemic work

Grids subdivide existing *shequ* and thereby seek to connect the local party-state with the local population (see Figure I). This connection is to be achieved in three ways: first, by the grid service team that consists of township and village cadres, police officials, teachers, community doctors, petition office representatives and locally respected individuals (Hu, 2013). Usually, three people lead this team – a grid chief, who is almost always also a member of the community party group, and two full- or part-time grid managers. While in some places, grid managers are recruited from within the grassroots bureaucracy, in other areas, grid members are employed through temporary service contracts (Interview 5). Second, each grid fulfils a range of functions for which a township-level leader is directly responsible, and thus held accountable through performance evaluations. Third, grids rely on digital tools and databases to collect and analyse population data.

**Figure 1.**
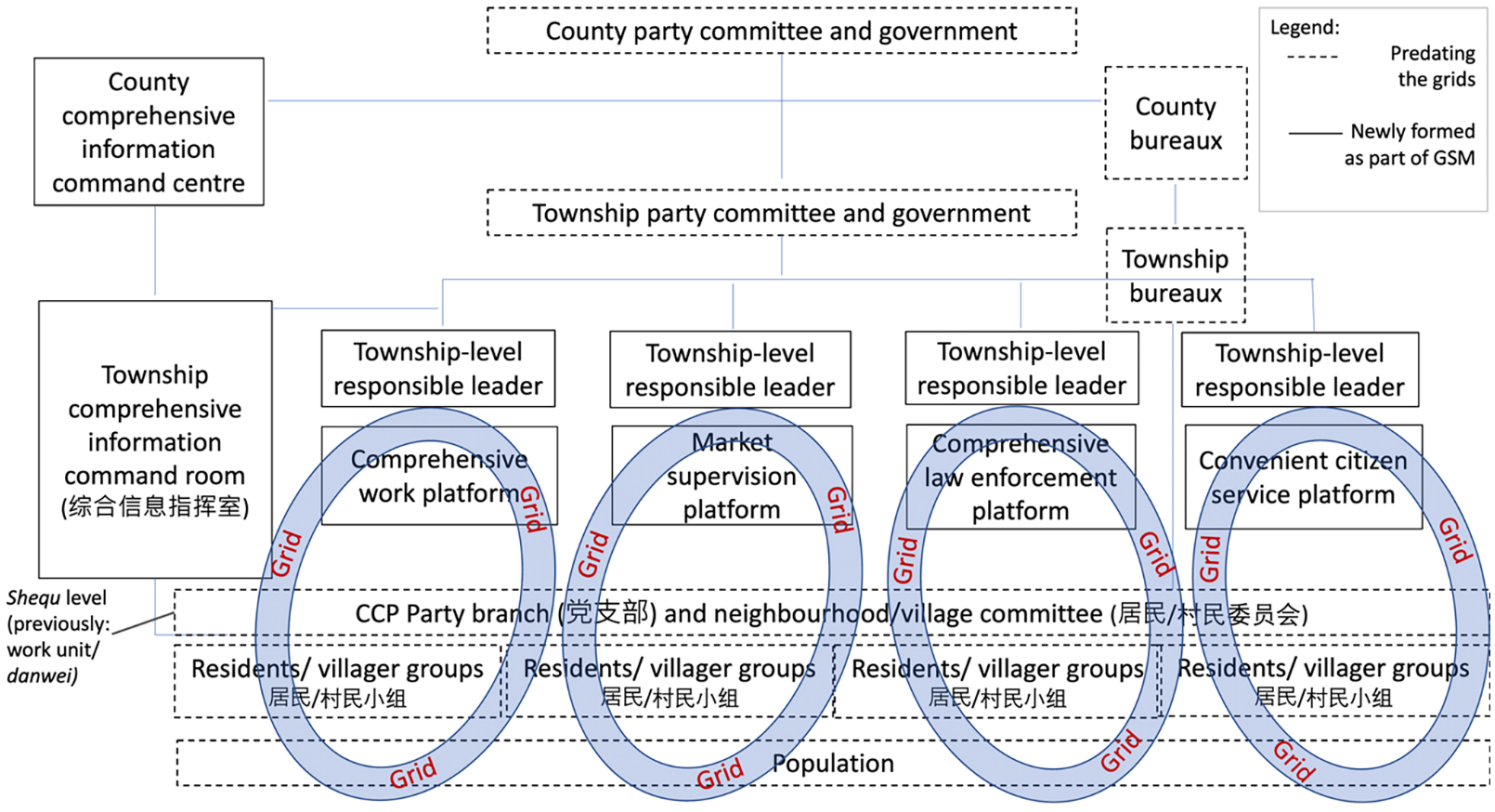
Grids within Fengqiao Township’s system of local governance. Source: Authors. Based on a board displayed in the township government building.

Grid managers’ most crucial task is to patrol the grids regularly (around twice a day), talk to residents and identify and solve potential problems in the community. All of the information collected during these patrols is noted down in a work diary, and problems that can only be solved with the help of higher administrative levels are reported upwards through a web application (Interview 3).^4^ Apart from gathering information through patrols, grid managers have access to a local database that provides them with information about individual households and population statistics (employment, housing, social security, family planning and economic development). This digital structure is supplemented by a physical network of offices that pool public resources to provide targeted services to grid residents ranging from cultural activities to food distribution and employment support (CSSN, 2013; Hu, 2013). In all of these tasks, grid workers rally volunteers to assist them.^5^

A grid manager from Chengdu describes her job as ‘having to ensure safety in the grid, to find suitable volunteers for different tasks, and to more generally promote the self-government of residents’ (Interview 3). This is in line with Palmer and Winiger’s observation that in China’s neosocialist governmentality, the party-state encourages ‘grassroots initiatives and volunteering, co-opting groups that train people of low *suzhi*^6^ to enhance their capacity to self-manage in the market economy’ (Palmer and Winiger, 2019: 571).

To show how sovereignty, discipline and security interrelate in GSM, the following sections investigate how grids have deployed these three forms of power during different times in the COVID-19 pandemic defined by their primary political objective, namely fighting the virus and fighting economic decline, respectively.

### The emergency phase: Closing the grid to fight the virus

In many localities, the initial COVID-19 crisis response by central government mainly consisted of direct interventions dictating local lockdowns and emergency response levels (Xu and Yang, 2020). Later, responsibilities and authority were delegated to over four million grid managers who police over 650,000 communities across the country (State Council, 2020). For example, in Zhuji, a county-level city in Zhejiang province, the emergency response measures were announced on 4 February 2020, referring to a list of ten items, most of which were also applied internationally, such as the identification, isolation and effective treatment of infected individuals, and the prevention of public and private gatherings. In addition, these measures included the closure of all public facilities, the screening and registration of all cars and passengers entering Zhuji, and the stipulation to wear masks in public. Landlords had to halt all rental business and were required to inform tenants from outside Zhuji not to enter the city (*CCTV News*, 2020).

In line with the general effort of the CCP throughout the reform period to shift the responsibility for social management to grassroots officials, grid personnel stood at the centre of implementing virus control measures. The listed responsibilities for grid managers included: (1) quarantine management, including keeping close contact with quarantined residents, making daily calls or video visits to potentially infected residents, supervising the temperature of quarantined residents as well as solving their daily needs, and providing psychological comfort; (2) procedural regulations including dividing responsibilities among the team and specifying procedures, e.g. for returning residents; (3) defining hygiene standards for communities, including disinfecting and sanitation services and ensuring the measures were accepted by the community; (4) controlling entry and exit to the grids, patrolling the grids, taking people’s temperatures and reporting all related information (including instances of non-compliance) to the district (*CPC News*, 2020; Interview 2; Interview 3; Interview 4).

Within each grid in Zhuji, grassroots party organizations and grid managers mobilized residents to become so-called civilian emergency rescue volunteers. Volunteers assisted in shutting down crowded places such as senior citizens’ activity rooms and street markets; helped raise scarce supplies and donated epidemic prevention materials; distributed masks and disinfectants; stood at the city’s highway entrances to identify passing cars which had entered Zhuji from high-risk provinces such as Hubei; undertook door-to-door calls to inform households about the virus and related anti-pandemic measures; and provided 24-hour psychological counselling services via telephone (Zhuji Daily, 2020a). Local doctors joined in as volunteers to take people’s temperatures and collect health information from all households within each grid (Zhuji Daily, 2020b). The collected data was then supposedly reported to a command room responsible for redistributing relevant information to four township-level platforms (see Figure I).

Together with the grid managers, volunteers played a dual role of control and care work, depicted in the slogan: ‘grid is both management and service’ (*Beijing News*, 2011). On social media, grid managers and volunteers were often portrayed as selfless and caring community members who put the well-being of others before their own. The slogan ‘to guard the community is to guard your own home’ emphasizes the goal of creating and making use of personal relationships in epidemic prevention and grassroots social management. Grid managers are supposed to consider the community as their family, and volunteers should take pride in working their fingers to the bone (Chengdu government, 2020). These images also manifest in how grid managers speak about their work. Although referring to their job as ‘security work’, they strictly differentiate their job from that of police officers, as grid managers

not only patrol the grid on the lookout for potential problems but also make sure to come into contact with the people, to become familiar with their situations and identify problems early on. When someone is sick, [they] organize volunteers that visit the person, help with a haircut, clean up their house, and so forth (Interview 3).

These examples show two things: first, grids are built upon trusting relationships – newly established and existing ones – that diminish private spaces as these become regulated and policed by community members. In doing so, the Party utilizes personal relations to appear close to its people and their perceived security needs. This governmentality deploys a ‘people-oriented’ type of security by positioning its agents as caring, virtuous and part of the same family (Dutton, 1992: 85f). Second, the self-responsibility of grid managers and the community at large is at the heart of how the grids function. During pre-crisis times and after the emergency phase, grid managers are encouraged to solve all problems within the community by themselves; help from superiors should only be sought when no solution can be found within the grid. During the emergency phase, grid managers and volunteers are tasked with reducing the number of infections within their grids and are granted considerable flexibility to identify ‘appropriate’ and effective measures to do so (Interview 4).

Grid managers were also pivotal as agents of the state and interpreters of upper-level regulations. In Xi’an’s Beilin district, for example, grid managers decided to use an app with a colour-coded system to indicate each resident’s compliance with quarantine regulations. The system assigns red (for not reporting back within three hours or not answering the phone), yellow (for people who have not passed the community review yet, but have been tracked by telephone) and green (for those who have passed the community review and have undergone home quarantine). Responsibilities for the different categories of residents are administered accordingly: residents marked as red are within the responsibility of the public security bureau, while people labelled as green or yellow are under the purview of grassroots self-management (*Tencent News*, 2020).

In sum, during the emergency phase, all three forms of power were deployed through the grids to create artificial barriers and make them manageable as milieux. First, sovereignty was the primary form of power deployed through lockdowns with almost impermeable grid borders for all citizens and home confinement for infected and potentially infected individuals. These techniques of sovereign power also included rules that stipulated punishments in case of any transgressions. Second, within these closed borders, disciplinary power was deployed through door-to-door calls and WeChat messaging to check on people and educate them about the virus and anti-pandemic measures. Discipline also worked on grid managers and volunteers trained through various means to take responsibility for any problems within the grid and treat grid inhabitants like their own family. While this technique of giving responsibility to grassroots staff was also present in later crisis times, the pressure put on grid managers by higher-level officials and the discursive framing of grid managers as servants for the community was most intense during the initial lockdowns across China (Interview 3). Finally, security was deployed through the collection of information by grid managers during their patrols and through WeChat messaging with residents. This information was collected in the work diaries of grid managers and was entered into township-level digital platforms used to supervise the epidemic situation and calculate the level of risk that each grid and its residents posed to public health. During the initial emergency period, security power – in contrast with discipline and sovereignty – was less dominant because risk levels and algorithms to predict risk as central elements of security power had not yet been established. Both of these elements became dominant during the second phase of the crisis when the primary purpose of the power mix was to reboot circulation rather than halt mobility.

### The twin wars: Reopening the grid to fight economic decline

In most places, the focus on ‘emergency response measures’ shifted towards resuming work and production after only a few days. In Zhuji, for example, the first official document signifying the shift away from a complete lockdown towards ‘controlling the epidemic and stabilizing production’ was published on 6 February. As stated by the document, the new goal was to minimize infections and simultaneously achieve previously set annual economic growth targets (*Zhejiang Daily*, 2020). In the following days, the first two highway entry and exit points and transjurisdictional buses to and from Zhuji resumed, and infrastructure projects and factories slowly restarted operations (*Zhuji Daily*, 2020c).

Many of the factories along China’s east coast depended on migrant workers who had returned to their home villages during the Chinese New Year festival and the subsequent lockdown. A nationwide survey among private small- and medium-sized enterprises conducted shortly after this period of reopening in March 2020 showed that about 40% were facing labour shortages at the time (Li et al, 2020). The resumption of work thus posed a logistic challenge to Zhuji’s factory managers, who contracted buses from all over the country to bring employees back to work in Zhuji. In this, they were supported by local government bureaux such as the Human Resources and Social Security Department of Zhaotong City in Yunnan province, which chipped in poverty alleviation funds to pay for migrant workers’ high-speed train tickets. Once migrant workers arrived in Zhejiang, Zhuji’s Municipal Bureau of Human Resources and Social Security arranged buses to form what the local media referred to as a ‘Resumption of Work and Production’ convoy, which picked up migrant workers from Hangzhou East Railway Station and drove them back to Zhuji (*Zhuji Daily*, 2020c; *Zhuji Daily*, 2020d).

As economic activity restarted, the grids, which during the first phase of emergency measures had been responsible for halting any flow of people, were now tasked with strictly controlling the slow resumption of movement. Grid managers had to establish a mechanism that allowed as many working people as possible to cross grid borders and resume work. At the same time, this mechanism was still required to detect people infected with the virus and those running the risk of becoming infected. In Zhuji, the process was divided into three phases: in the preparatory phase, the city government informed businesses about epidemic control measures, requiring grid managers to inspect the implementation of these measures and approve or decline factory reopenings. In the return-to-work phase, grid managers registered to return migrants and other workers and managed their quarantine. In the work resumption phase, work in the factories restarted, and grid managers were tasked with monitoring the factories’ adherence to epidemic prevention practices (Zhuji government 2020).

During this time, when daily reported infections were low, the number of volunteers supporting the grids was reduced. Emergency response measures were replaced by ‘institutionalized and standardized management and control’, also called ‘intelligent control’. City officials in Zhuji, for example, were called upon to start tracing infections through a combination of ‘big data and grid management’, which allowed them to assign responsibility for epidemic prevention to the grassroots level and establish digital indicator systems through which to trace infections (*CCTV News*, 2020). A so-called ‘precise intelligent control index’ was used to create epidemic maps based on the number of infections in individual townships and colour-code regions according to risk levels (*Dushi Kuaibao*, 2020; Xuexi Qiangguo, 2020).

The first local health code system was launched in Zhejiang in mid-February 2020. Developed by consumer technology companies Alibaba and Tencent, users access the health code through one of the companies’ apps, Alipay or WeChat. After registering with their phone number, full name and ID number, the app uses shopping, travel and medical data to assign users a red, yellow or green QR code. A green code grants users access to public spaces, while a yellow code indicates a potential contact with an infected person requiring a seven-day home quarantine. A red code supposedly identifies users infected with the virus, stipulating a 14-day quarantine (Chen, 2020). While grid managers were responsible for controlling the QR code of everyone entering or exiting the grid, the use of the health code to cross grid borders fluctuated over the course of 2020, depending on the perceived regional urgency of epidemic prevention (Interviews 1 to 5). The colour codes not only provided information about users’ health conditions and levels of compliance with pandemic measures but were also supposed to facilitate data transfer between community databases and higher administrative levels (Liang, 2020). Although not technically integrated with social credit databases, local governments such as Hangzhou have begun to punish individuals’ false reports by deducting their credit score and publicly blacklisting them on the Credit Hangzhou website (Credit Hangzhou, 2020).

Health-code systems were implemented all over China but with local variations. In Yunnan province, the Civil Affairs Bureau of Lijiang County formulated a ‘six-nets programme’ that also included the enforcement of a ‘Yunnan epidemic’ online system built on QR code tracking. Until mid-February 2020, the city had established 17,478 codes for people to scan when entering or leaving a building (Ministry of Civil Affairs of the PRC, 2020). In Shanghai’s Baoshan district, the health code was provided via a local online platform called community pass, a community governance platform that combines mobile internet, Big Data analysis and other technologies to collect mobility data. The community pass allowed residents to take an ‘express lane’ upon entering the city to work. Responsible for the collection and verification of information, grid managers appointed epidemic community workers who randomly phoned residents to verify the information they had provided to the platform, constituting an additional layer of policing (Cyberspace Administration of China, 2020).

In Changzhou, a prefecture-level city of Jiangsu province, automated health checks were conducted daily with every resident. This was done through facial recognition systems installed at community gates, which automatically measure body temperature. Facial recognition also lets residents enter without keys, preventing non-community members from entering. A community manager in Nanjing, where a similar technology was applied, was quoted as saying ‘These new technologies have reduced the burden on grassroots epidemic prevention work and enhanced grid governance capabilities. The community is also more flexible in its personnel deployment and can free up more energy to investigate dead ends.’ (Cyberspace Administration of China, 2020).

Aside from these positive portrayals by government agencies and state media, numerous personal accounts on Chinese social media reveal that the health code was working inconsistently, especially in its early stages. People used the online government platforms to complain about unreasonable lockdown measures that had been implemented as a result of malfunctioning health codes (Message Board for Leaders, 2020).

In sum, in line with what Foucault describes as a shift from the individual to the population (Foucault, 2009), in this second stage of epidemic control at the grassroots level in China, the focus shifted from micromanaging individuals’ health and quarantining individuals towards managing the reopening of the economy. With the goal of fighting economic decline (while controlling the virus), this phase saw an increase in data collected and analysed to determine overall levels of risk emanating from the reinitiated flow of people and the implementation of targeted lockdowns.^7^ During this time, all three forms of power were deployed through the grids – albeit each in varying intensities and with different instruments. In contrast to the first stage, which saw virus elimination primarily through sovereign and disciplinary instruments of power, the second stage shifted towards reviving circulation primarily by security instruments bristling with discipline and sovereignty.

First, sovereign power was toned down as grid borders became permeable for those with a green QR code. For these citizens, the border did not represent an instrument of sovereign control. However, their mobility was regulated by sovereign means, which stipulated who was allowed to travel and which quarantine and testing measures they had to undergo. For individuals with yellow and red codes, sovereign power remained the same, as they were still subject to strict quarantine regulations. Hence, in contrast to the first phase of grassroots epidemic management, sovereign power was more selective in its targets and less visible to an increasing number of people. Second, disciplinary power deployed on grid residents and grid workers (including volunteers) was also reduced. Grid workers were no longer required to conduct daily door-to-door calls or WeChat messaging. Rather than personally informing residents about how to behave during this process of renewed circulation, grid managers switched to impersonal means of communication such as posters in residential compounds. Disciplinary instruments of power thereby became more diffuse and less interventionist.

Finally, security power was increased by equipping grids with digital means to collect and analyse data.^8^ Throughout the phase of resumed mobility, the health code became the central tool to guide the circulation of people inside and beyond each grid (*Zhuji Daily*, 2020c). In doing so, the health code functions as an instrument of security, discipline *and* sovereignty. It represents sovereignty as a border pass that allows users to move between grids. It acts disciplinarily by reminding people that only ‘necessary’ (or state-sanctioned) mobility should be undertaken to reduce contact and risk of infection (or a red QR code). It is an instrument of sovereignty through its collection of data on individuals and its authority to turn them into colour codes interpretable by grid personnel.

## Conclusion

This article discusses how the handling of the COVID-19 pandemic in 2020 reveals the continuous efforts by China’s party-state to govern through spatial technologies of social management. In contrast to prior studies on the grids, which have scrutinized the process of policy design (Mittelstaedt, 2021) or evaluated grid governance based on the original goals laid out during the policy process (Xu and He, 2022), we analyse GSM from a spatial security perspective that regards grids as tools which deploy different forms of power mixes across time and space. Understanding grids as topologies of power helps us to differentiate how sovereignty, discipline and security work in tandem while prioritizing diverse mobility strategies throughout different crisis phases. We show that China’s neosocialist governmentality builds on spatial tools to regulate (im)mobility and circulation, fixity and fluidity among different populations. Through this, the party-state can anticipate various events but simultaneously establishes mechanisms that allow for disciplinary action when necessary.

This flexibility of China’s spatial security apparatus has become ever more prevalent during the COVID-19 pandemic when existing grid structures were employed first to halt the spread of the virus and then to restart economic activities. Hence, rather than speaking of a period of potential ‘reorientation’ of GSM during the pandemic (Mittelstaedt, 2021: 18), we show how grids have become instruments that flexibly reorient themselves in line with current political or crisis modes the political system finds itself in. While we show that this process of flexible reorientation has progressed smoothly and has most likely contributed to a decrease in infections over our study period, we did not set out to evaluate the overall effectiveness of GSM in terms of its original policy goals or potential negative consequences. Accounts of selective and distorted GSM implementation are increasing (Xu and He, 2022; Li, 2022), underlining a well-known phenomenon of central–local relations in China.

Instead, we aim to show how instruments of spatial control in China regulate mobility by deploying different forms of power. We show that to keep up with the circulations of population elements through the grids, Chinese neosocialist governmentality more generally builds both on the ‘informationalization’ of citizens (i.e. making individual behaviour transparent and available to the government through the collection of data) and the self-regulation of communities, and both of these may be used as instruments of discipline and security. While GSM has only been developed during the reform era, it builds on a long history of gathering and saving data (on people’s family structures, income, health, fertility, productivity), on the internalized acceptance among Chinese citizens of government intervention in their private lives and on making subjects ‘manageable’ by organizing them according to levels of risk that they purportedly pose to state projects.^9^ During the pandemic, grid managements appropriated existing data collection forms, drawing on comprehensive data about residents, including travel histories, health data and family structures.

Second, new digital technologies such as health codes play a crucial role in the technical implementation of pandemic measures and the spatial reproduction of the security system. Besides undertaking risk assessments for the government, data analysis legitimizes government decisions by informing them with ‘scientific’ information and creating the impression of ‘intelligent’ (or ‘modern’) epidemic control. At the same time, the diversity in local applications of the health code system and the implementation of grid management more generally has produced fragmented and digitized spaces of security. This fragmentation is due to differences in local grid structures, health code algorithms and the new authority of grid managers and volunteers as data managers that determine ‘appropriate’ reactions to rising infection numbers and define what is considered ‘normal’ or ‘safe’.

Third, through regular contact with grid personnel, GSM is designed to ensure that residents identify with their local community, ultimately creating a security apparatus that permeates private and public spheres. This identification is reinforced by recruiting trusted community members into the security apparatus. Senior community members are co-opted as grid volunteers linking personal trust to party politics. While the state defines the realms of responsibility for each citizen in relation to the grid structure, the grid management manages ‘critical mobilities’ and punishes risky individuals accordingly. This ultimately co-responsibilizes the community, increasing regime support by empowering community self-government (Heberer and Göbel, 2011; Qian and Hanser, 2021). At the same time, integrating each individual into a local system of mutual responsibility creates ‘active’ citizens, mobilizing society rather than creating barriers.

Furthermore, we wish to emphasize that we do not regard the security apparatus as ‘perfect’. The above-mentioned online complaints about health code implementation or the public outcry about authorities misusing the health code to prevent public protest against corrupt bankers in Zhengzhou (Henan province; CNN, 2022) show how people resist, challenge and counter official narratives. On the other hand, it could be argued that the system’s imperfection is part of the security apparatus’s design to make users even more cautious when travelling, forcing them to negotiate their behaviour with the ‘responsible’ grid manager. Ultimately, our investigation presents a timely example of how the Chinese state responds to, manages and is willing to subscribe to the notion of crisis. It shows how the grid as a flexible tool for regulating circulation in non-crisis times also functions as an instrument for crisis management.

By integrating sovereignty, security and discipline into community self-control, GSM allows human and financial resources to be mobilized in case of health and other emergencies (Thornton, 2009: 28). The flexible application of GSM after the initial lockdown successfully rebooted the economy, and thus legitimized the system. During the Central Economic Work Conference in December 2020, the CCP emphasized that ‘scientific decision-making and creative responses are the fundamental methods to turn crisis into opportunity’ (Xinhuanet, 2020). As such, a crisis is not perceived as a government failure but as ‘coterminous with the wider political milieu that produces it’ (Jeandesboz and Pallister-Wilkins, 2015: 317).

## Notes

1. China’s multilevel administration system is divided into five hierarchical levels of government: the central, provincial, prefectural, county and township levels. Depending on the level of development of the regions in which township-level governments are located and whether they are in an urban or rural area, the township level includes town governments, street-level administrative offices or township governments. Villages and communities (*shequ*, urban counterparts of villages) are officially referred to as self-governing units outside the domain of China’s system of local government (Heilmann, 2017).

2. Due to the visibility of the case in local media and the ability of our informants in China to comment and contextualize the case, our examples often draw on GSM in Zhuji. Zhuji is a county-level city in Zhejiang province located on China’s east coast. While it is more affluent than other parts of China and relies heavily on internal migrants to staff local factories, it is a particularly illustrative example of how GSM shifted between different topologies of power.

3. Similarly to the *hukou* household register, the work unit *danwei* functioned as a unit of welfare provision within the Maoist production system (Bray, 2008: 396).

4. In Dongcheng district, officials claimed that the number of complaints decreased after the introduction of GSM (*Beijing News*, 2011).

5. Grid volunteers are mostly senior residents who, after retirement, substitute their previous work lives with social activities within residential communities (Tang, 2020: 53).

6. *Suzhi* is usually translated as population ‘quality’ as a proxy for the economic and social value of individuals for the society (Sigley, 2009).

7. Single infections could lead to closures of several communities with thousands of residents, each of whom had to undergo a Nucleic Acid Amplification Test (e.g. Xinhuanet, 2020).

8. While information was collected through the health code and the web applications that grid managers use to pass on their information to local databases, data analysis was mostly done at the county level (Interview 5).

9. Due to spatial constraints and the complexities of these systems, we cannot provide a systematic comparison at this point. Further research should examine how the systems of GSM and *hukou* correlate and whether we can speak of a genealogy of spatial governmentalities in China. The different subjectivities of the systems argue for that genealogy. While the *hukou* system aims to govern productive populations, the grids govern productive communities. Both methods build on registers that assign citizens a status in which the government evaluates their potential risk for a productive society. In the *hukou* system, local governments assess this risk, whereas in GSM, this assignment relies on the community cooperating with the grassroots party-state.

## Interviews cited

Interview 1: Community volunteer from Hubei province, 23 October 2020
Interview 2: Community volunteer from Hubei province, 29 October 2020
Interview 3: Grid manager from Chengdu municipality, 22 December 2020
Interview 4: Grid manager from Guizhou province, 23 October 2020
Interview 5: Professor from Central Party School, Beijing, 15 November 2020
